# Semimembranosus: A Rare Muscle Herniation and Review of the Literature

**DOI:** 10.7759/cureus.40001

**Published:** 2023-06-05

**Authors:** Göker Utku Değer, Baris Gorgun, Soner Koçak, Veli Muzaffer Murat Hız

**Affiliations:** 1 Orthopedics and Traumatology, Beykoz State Hospital, Istanbul, TUR; 2 Pediatric Orthopedics, Ortopediatri Istanbul Academy of Pediatric Orthopaedics, Istanbul, TUR; 3 Orthopedics and Traumatology, Kanuni Education and Research Hospital, Istanbul, TUR; 4 Orthopedics and Traumatology, İstanbul University - Cerrahpasa, Cerrahpasa Faculty of Medicine, Istanbul, TUR

**Keywords:** literature review of disease, prolene mesh, lower limb reconstruction, focal muscle herniation, muscle injury

## Abstract

Extremity muscle hernias are rare pathologies, most of which are managed conservatively. In symptomatic cases, surgical intervention may be required. This study represents a case of a rarer muscle hernia, semimembranosus, in a 43-year-old patient and describes the surgical technique of grafting with synthetic nonabsorbable polypropylene surgical mesh as well as the review of the literature about extremity muscle hernias.

## Introduction

Extremity muscle hernias are rare pathologies in which the muscle protrudes outward as a result of a defect caused by a congenital pathology or past trauma on the muscle fascia. Most of these cases are managed conservatively; however, surgical treatment is preferred if they become symptomatic. Among modern surgical techniques are primary repair, fasciotomy, autologous fascia lata reconstruction as well as alternatives such as grafting with synthetic meshes [1-4]. The tibialis anterior is regarded as the most frequently herniated muscle in the literature, as it is subjected to trauma more frequently [3-5]. In diagnosing muscle hernias, which are provoked especially as a result of extremity motion and become visible, ultrasonography (USG) is the preferred method because it is a dynamic imaging technique [6-10].

This case study will focus on the surgical technique used for the repair of the semimembranosus muscle hernia with synthetic mesh, as well as the review of the literature on muscle hernias. Informed consent had been obtained from the patient for the study.

## Case presentation

**Figure 1:**
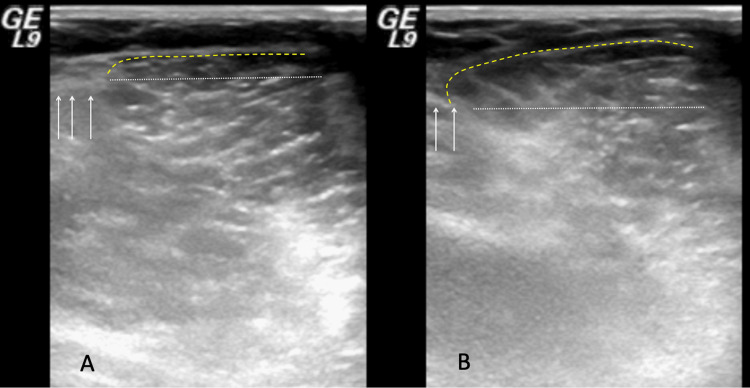
Ultrasonographic evaluation of the fascia defect both in relaxation (A) and contraction (B)

**Figure 2:**
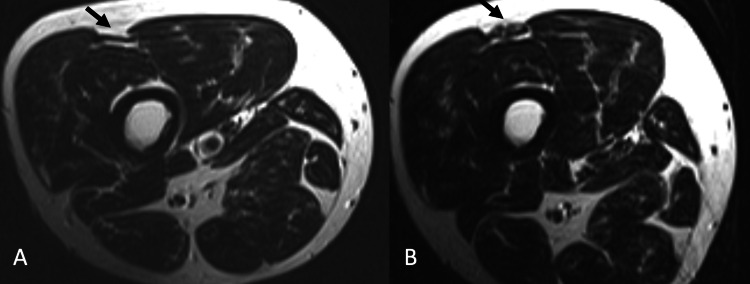
Dynamic MRI evaluation of the fascia defect showed with arrow both in relaxation (A) and contraction (B)

A 43-year-old male patient who was admitted to our department with complaints about the swelling on the right side of the distal posterior thigh of which he had been aware for the past 20 years, and recently accompanied by pain, had been working while standing for prolonged periods of time as his profession required him to do so (a waiter). Upon physical examination, a 1.5 cm, mobile, soft-consistency mass was detected on the right distal posteromedial thigh. It was observed that the mass became apparent while flexing the knee. The dynamic USG revealed a defect on the semimembranosus muscle fascia as well as the muscle protruding through the defective area during motion (Figure 1). Whilst no pathology was detected on the MRI performed in a resting position (Figure 2a), the MRI that was performed while the muscle was contracted revealed that the semimembranosus muscle protruded toward the skin (Figure 2b). Surgical repair with synthetic mesh was planned for the patient diagnosed with a semimembranosus muscle hernia.

**Figure 3:**
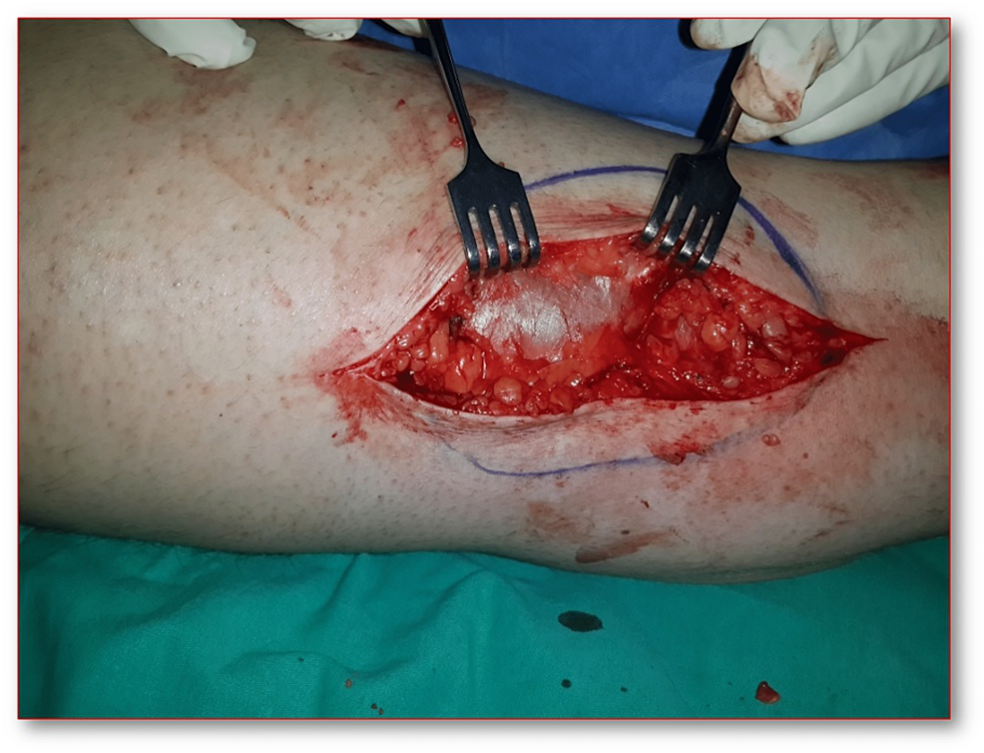
Surgery photos of the fascial defect

**Figure 4:**
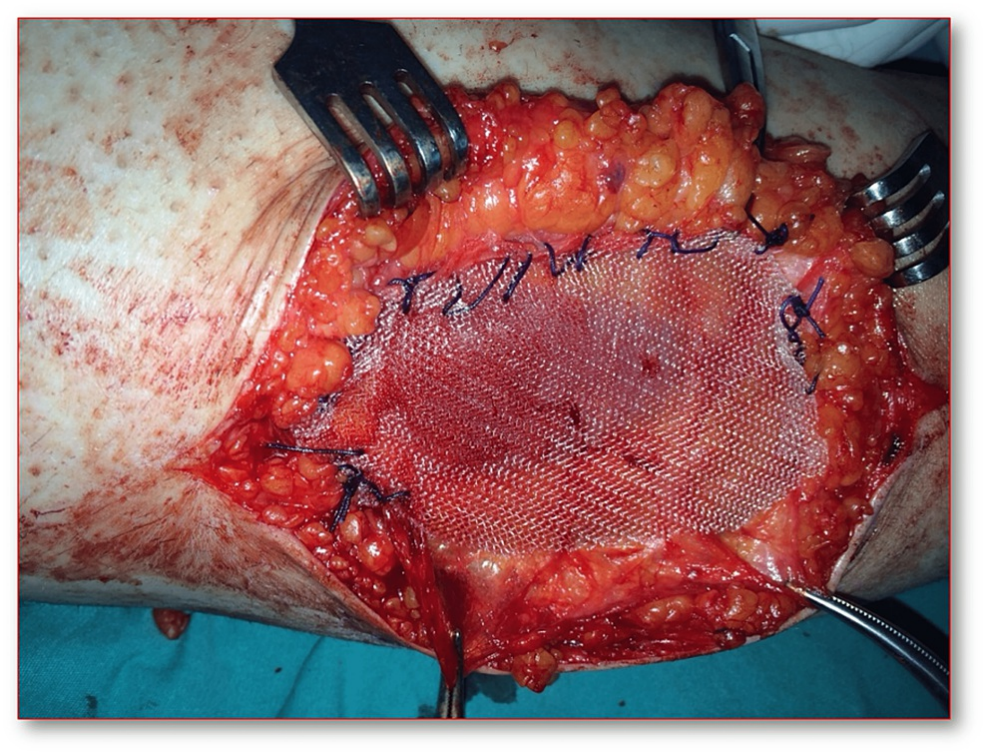
Surgery photos of the fascial defect after grafting with synthetic mesh

Surgical operation was performed through the 10 cm longitudinal incision immediately above the area with the fascial defect on the femur distal posteromedial, which had previously been marked with USG on the patient while in the left lateral decubitus position. Once the fascial defect was explored, the defected area was sutured to the surrounding undamaged fascial tissue with synthetic absorbable sutures (No:1 round vicryl, ETHICON^©^; Raritan, New Jersey) using appropriate tension with synthetic nonabsorbable polypropylene surgical mesh (8x3 cm prolene, ETHICON^©^) (Figures 3, 4). No weight-bearing was applied on the right extremity of the patient during the postoperative three weeks. Following the range of motion exercises, the patient returned back to his daily routines six weeks postoperatively. No infection or foreign body reaction was observed after the surgery. It was observed during the postoperative third-month visit that the patient had no complaints, nor any pain or swelling with movement. The VAS value, which was 80 prior to the operation regressed down to 30 after six weeks and 0 after three months postoperatively. Five years after the operation, it was confirmed in a telephone conversation that the patient resumed his active work life without any complaints.

## Discussion

Focal muscle protrusion through muscle fascia, which has weakened or lost its integrity is considered as muscle hernia [11]. Although it can be seen in the upper extremities, it is more common in the lower extremities [12]. Much as it is seen among athletes as a result of exposure to trauma and excessive activity, it has also been reported for occupational groups with excessive activity such as the patient in our case. Although muscle hernias were considered rare pathologies in the past as a consequence of dynamic methods for diagnosis not being preferred as well as the fact that the disease is asymptomatic, an increase in the number of these pathologies is being reported in the literature in parallel with the advancement of the dynamic diagnostic techniques [5,6,10,11,13]. Muscle hernias were categorized into two groups for the first time by Ihde in 1929 as structural and traumatic [10]. Structural muscle hernia is when the muscle protrudes the weakened muscle fascia, especially after chronic stress. Trauma-related muscle hernia can occur both as a result of a direct penetrative injury or a closed fracture fragment damaging the fascia, as well as an impact suffered by a contracted muscle resulting in an indirect trauma [2]. Furthermore, muscle hernias that are secondary to chronic compartment syndrome or develop postoperatively may be seen [14]. Chronic effort-related compartment syndrome generally affects military personnel, mountaineers, skiers, and athletes. Much as herniations have been reported in the literature most frequently on the tibialis anterior muscle as a consequence of it being prone to trauma, they have also been reported for the other lower extremity muscles; in particular, the extensor digitorum longus, peroneus longus, peroneus brevis, gastrocnemius, and hamstring muscles [3-5,15]. In addition, muscle hernias should also be considered among potential causes of inexplicable pain or inflammation in extremities even without a traumatic history.

 A muscle hernia can be considered a palpable soft mass lesion or nodule in clinical assessment. It can be singular or multiple and is usually unobservable while the muscle is in a resting position. Patients are usually admitted to the clinic with complaints such as pain, weakness, cramp, and numbness seen, especially post-activity. Infection, varicose veins, vascular malformations, and especially neoplasms should be excluded from the distinguishing diagnosis of muscle hernias [16]. The most significant feature of muscle hernias that distinguishes them from these pathologies is that the pain frequently disappears with rest and resurfaces with activity. Keeping muscle hernia in mind during the distinctive diagnoses of mass lesions can be considered an effective precaution against the need for additional invasive procedures such as biopsy and the period in which the patient would be affected psychologically as a result of it failing to be diagnosed in the future. Since the protrusion of the muscle through the fascia defect is more apparent during movement, dynamic imaging methods should be preferred in radiological examination [2]. Although the majority of literature supports the view that favors the fact that this protrusion, which is caused by movement, generally happens during isometric contraction, Naffaa et al. reported that herniation will not always become visible with muscle contraction but will do so with contraction when the herniation on the longitudinal axis of the muscle appears on the body of the muscle, however, herniations that are in the vicinity of the muscle origin or insertion would be visible in resting position [9]. Despite the lack of a consensus in the literature regarding an imaging method, USG, with its ease of access, low cost, and especially dynamic examination capabilities, has been suggested as the gold standard method by many authors [6,8]. Çarlı et al. noted the user-dependent variability as the only disadvantage of the USG [8]. When diagnosing the herniated muscle without it being reduced, it is important to carefully apply the probe gently [17]. The standard MRG, albeit useful in excluding distinctive diagnoses, is insufficient in diagnosing muscle hernias [12]. Kramer et al. pointed out in their study that of 18 patients who were examined with MRG, only three were diagnosed with muscle hernia [1]. However, in situations where dynamic USG fails to identify the defect, it is recommended that dynamic MRG should be used for the identification of the size and structure of the defect or to better observe the muscle fascia demarcation [12]. Moreover, MRG is considered to be helpful for the surgical planning of muscle hernias.

Follow-up is sufficient for managing these cases, as long as the asymptomatic cases do not constitute an aesthetic concern. On the other hand, conservative methods (rest, elastic bandages, anti-inflammatory drugs, etc.) should be used for symptomatic cases [13]. Surgical treatment is recommended for cases that do not respond to conservative treatment [18]. Alternatives such as primary repair, fasciotomy, partial muscle excision, reconstruction with autologous fascia lata, or grafting with synthetic mesh are among the surgical treatment techniques [4,15,18]. Despite the fact that primary repair is known to provide successful restoration of small defects, the development of the compartment syndrome has made longitudinal fasciotomy the most recommended and safest technique [3]. However, Kramer et al. reported ongoing complaints post-fasciotomy at a rate of 53% in their study, which was conducted with 26 athletes [1]. For this reason, as well as potential negative cosmetic results, we did not favor longitudinal fasciotomy on this patient. On the other hand, the primary repair method is not recommended for defects such as the one present in our case, whose 4x3 cm fascia defect is deemed relatively large, especially if the ends of the fascia are strained since it is suitable for small defects and carries an important complication risk such as compartment syndrome [13]. Miniaci et al. reported a case that resulted in a drop foot as a complication of evolving and repetitive debridement, following the primary repair of the tibialis anterior muscle hernia, which led to a search for alternative treatment options [3]. Furthermore, as they cause morbidity in the donor area and extended the surgical duration, fascia lata or similar autografts are preferred less often. Favoring results have been reported in recent literature regarding the repair with synthetic mesh and acellular collagen matrix for massive defects and mobile patients [15,18]. Tarrant et al. showed in their study that repair that was performed by using an acellular dermal matrix showed biological compatibility and efficiency of results without any complications for four consecutive post-traumatic massive fascial defect herniation [19]. Due to the conditions of our clinic, grafting with a synthetic nonabsorbable mesh was preferred for our case. The relief of pain during the sixth postoperative week and the resumption of complete activity after the second month demonstrate that stability has been achieved and is coherent with the literature. The lack of recurrence i­­n the long term and the disappearance of the pain alongside the regained functionality have been consistent with similar studies [20].

## Conclusions

Although a conservative approach should be the preferred treatment method in symptomatic muscle hernias, surgical treatment options - especially for cases that do not respond to conservative treatment - should be kept in mind. Despite the lack of a consensus in the literature about an ideal surgical method, grafting with synthetic mesh has been shown to provide successful functional and cosmetic outcomes in lower extremity muscle hernias and be applicable in such cases.
